# How Science Can Perfect Fine-Flavor Chocolate

**DOI:** 10.1021/acscentsci.3c01191

**Published:** 2023-09-29

**Authors:** Rachel Brazil

Vijay Jagassar describes the cocoa produced at his Trinidad estate as “dark, woody, [with a] very strong chocolate flavor and minor floral notes.” In 2021, this intricate combination propelled his product to the finals in the Trinidad and Tobago National Cocoa Awards Competition.

Unlike bulk cocoa, which ends up blended into the bars produced by firms such as Hershey and Cadbury, Jagassar produces fine-flavor chocolate, which is sought out for its unique taste notes.

An engineer by training, Jagassar returned from Houston to his native Trinidad in 2018 and knew he wanted to take a more scientific approach to producing fine-flavor chocolate, so he contacted the Cocoa Research Centre (CRC) at the University of the West Indies. The Jagassar estate became one of the farms to participate in the CRC’s initiative to understand how cacao fermentation—a natural days-long process that converts raw cacao beans to velvety cocoa—creates the molecules behind chocolate’s flavor.

Cacao beans sold to produce fine-flavor chocolate fetch significantly higher prices: US$5,500–$6,800 per metric ton (t) versus $2,500–$3,000 for the bulk cocoa that makes up 90% of the market. Jagassar can sell a single bar of chocolate for £13 ($15.80) by using a “bean to bar” model, in which chocolate is produced using only his cocoa.

**Figure:**
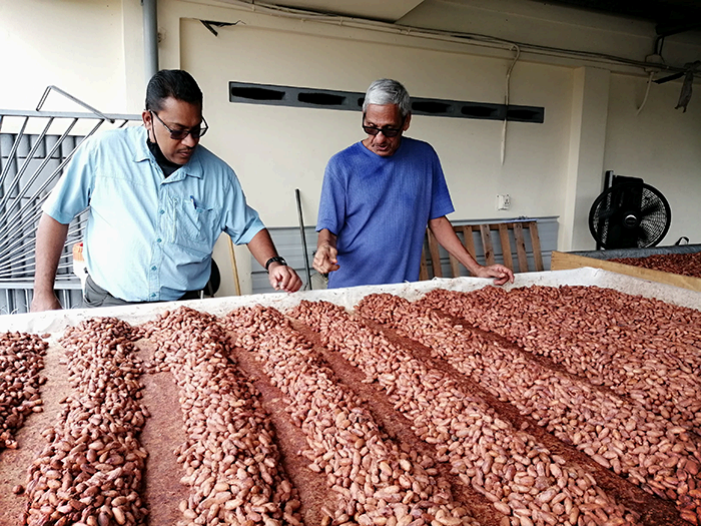
CRC research fellow Darin Sukha (left) and cacao farmer Ramraj Ramdial discuss sampling the beans on the drying floor at the Golden Beans Estate. Credit: Cocoa Research Centre.

And he’s not alone. The bean-to-bar market is expected to expand by 7.8% annually over the next 5 years, according to a report by research firm Mordor Intelligence.

The CRC project, which is part of a larger collaboration with the University of Nottingham and recently retired plant geneticist David Salt, involves farmers as citizen scientists at eight cocoa estates across Trinidad’s six agroecological zones.

As part of the effort, CRC researcher Naailah Ali worked with the estates in 2021, observing cacao bean fermentation on their estates. The farmers monitored the temperature and pH of the mounds of cacao during the fermentation and drying process.

They also froze bean samples and sent them to the CRC lab in Trinidad. There, researchers identified which types of microbes were working their magic during fermentation and turning the initially flavorless beans into the fruity, floral, nutty, creamy, or spicy notes of fine-flavor chocolate. At the end of the project, a sensory analysis of the final cocoa liquor—the paste made from the fermented, roasted, and ground cacao beans—characterized the flavors produced.

All these numerical and sensory data contribute to researchers’ growing understanding of the chemistry behind fine-flavor chocolate fermentation. One part of this work involves determining which molecules correspond to which sensory notes. From there, researchers are identifying and even trying to reverse engineer the networks of yeast and bacteria that manufacture those flavor compounds during fermentation. They envision a world in which farmers could pick and choose from a library of microbes and create a tailored flavor experience.

## Fine flavors in chocolate

At first glance, Irene Chetschik’s food chemistry lab at the Zurich University of Applied Sciences looks as if it pits human versus machine. In reality, the two are working together.

To start, Chetshik’s team vaporizes a chocolate sample and separates it into its component molecules. The compounds are sent down two separate paths.

One path leads to a mass spectrometer that does conventional analysis; the other leads to a sniffing port with a human assessor. The combination of techniques allows the team to identify compounds in chocolate that are responsible for creating the flavors that the assessor smells.

Chocolate is more complex than flavors that stem from a primary molecule, such as vanilla. “There isn’t a molecule in cacao beans that’s chocolate flavor,” Salt says. “The overall ‘chocolatiness’ is a bouquet of different compounds” that depends on the variety of the cacao tree, *Theobroma cacao*, and on the microbial communities supported by a particular local growing environment, or terroir, he adds.

**Figure:**
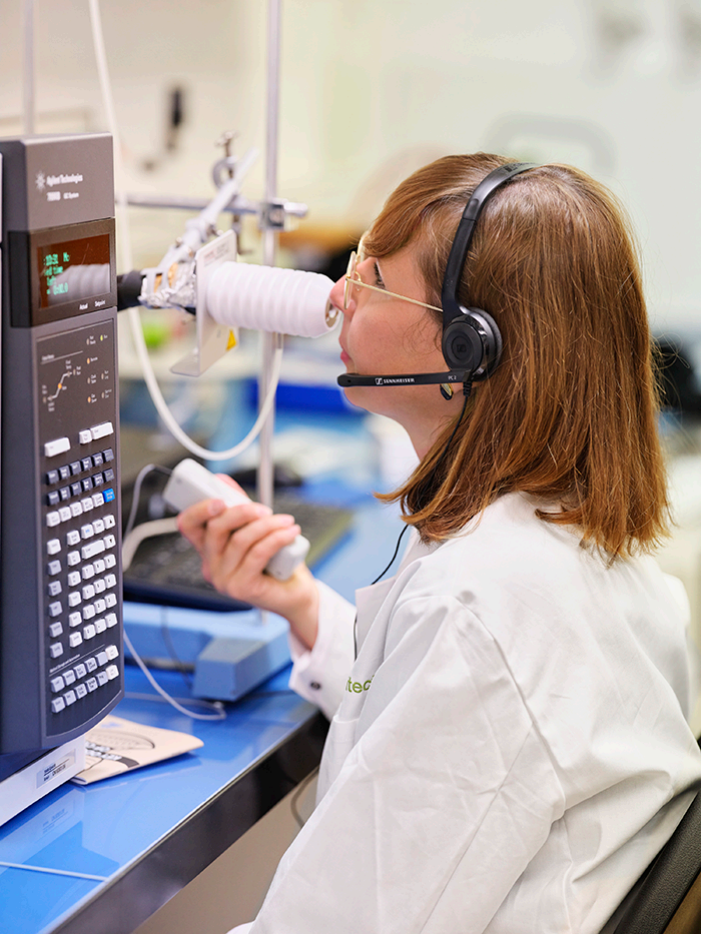
Food chemist Irene Chetschik analyzing the elements of chocolate’s aroma using gas chromatography olfactometry. Credit: Irene Chetschik/Zurich University of Applied Sciences.

Chetschik has been using the integrated mass spectrometry–human assessment approach—dubbed gas chromatography olfactometry—to probe the 100-plus volatile organic compounds (VOCs) detected in chocolate. Both machine- and human-derived data are vital because, even though some compounds affect the perceived flavor in a big way, they can be present at concentrations of less than 5 ppb. “You need to dig very deep analytically to get them,” Chetschik says, but a human nose can discern those flavors relatively easily.

Her lab has been able to identify some VOCs with characteristic flavors. For example, acidic and fruity flavors were associated with several esters, including ethyl 2-methylbutanoate and ethyl 3-methylbutanoate; roasty cocoa flavors with 2-methylbutanal, furanone, and dimethyltrisulfane; and floral and astringent flavors with polyphenol flavonoids.

The huge number of compounds Chetschik is finding explains the complex flavors that develop during cacao fermentation and why other researchers are finding fermentation so difficult to replicate or standardize.

## Precision fermentation

To some extent, chocolate connoisseurs have attached taste profiles to specific terroirs. The Piura valley on the western slopes of the Peruvian Andes is known for producing chocolate with floral and fruity flavors, while the humid region to the south of Lake Maracaibo yields product with a nuttier taste, says food technologist Carlos Hernández Aguirre, who studies cocoa fermentation at the National University of Costa Rica. (Hernández Aguirre usually goes for clean fruit acidity, floral aromas such as roses and jasmine, and tropical fruits and raisins.)

Each terroir comprises myriad environmental factors, including the local microbial communities crucial to producing the flavors that cocoa beans take on as they ferment. But studying these microbes presents a challenge. While work like Chetschik’s links flavors to individual molecules, it is still not possible to link a VOC directly to a single microbe species. This is partly because of the interconnected nature—not to mention the sheer diversity—of the microorganisms that break down cacao.

The basics are relatively well understood: fermentation starts with yeasts that are found all around us. They produce ethanol by breaking down sugars in the pulp that surrounds the beans in cacao pods. Bacteria consume the ethanol and produce lactic acid, which in turn is consumed by other bacteria that convert it to acetic acid.

Heat emanates from the beans as this chain of bacterial digestion progresses. That temperature boost and the acidic conditions destroy the cell walls of the cacao bean, thus allowing the breakdown of the proteins the cells contain and of cacao’s astringent polyphenols. All these compounds are then swept into a crisscross of metabolic pathways, which modify the small molecules bit by bit until cocoa producers arrive at that complex-yet-familiar chocolate taste.

But fermentation is a delicate dance. Leaving cacao too long leads to acid buildup and rancid or vinegar notes.

The Nottingham-CRC collaboration has sought to understand and harness the wider microbial communities that work in concert to produce particular flavor profiles. In addition to collecting samples from farmers in Trinidad, the researchers worked with farmers in Colombia, where they used a portable DNA sequencer in the field to catalog microorganisms present in the samples.

The team is now trying to understand and model the relationships between the hundreds of different species it has sequenced by connecting the actions of microbes that occur together—essentially reconstructing mini ecosystems of up to 30 strains that depend on each other.

In Nottingham, scientists have built a library of 500 microbes and are testing fermentations using smaller groupings that their analysis has shown work together to produce cocoa flavors. Once this system is validated, they plan to add or remove microbes from each group to bring out certain tastes. Salt calls the approach “precision fermentation” and says he is hopeful it could provide the full story of which microbe networks can produce which flavors. The team is hoping to publish its results soon.

But other researchers, such as Hernández Aguirre, are looking for a quicker solution. His team is focusing on the yeasts that provide the crucial jump-start in the fermentation process. The researchers need to optimize this early phase of fermentation to set the stage for the rest of the microbial community to flourish and develop the desired flavors.

With collaborators in Belgium, Hernández Aguirre has carried out several studies using yeast isolated from spontaneous cacao fermentation. In their most recent study, they compared two yeasts and found that *Saccharomyces cerevisiae*—brewer’s yeast used to improve flavor in beer and wine—produced cocoa liquor with a richer and more reproducible aroma. The second natural yeast they tested could not outcompete other microbes and was unable to establish itself, which led to underfermentation and more bitter polyphenols.

“Right now we are working on scaling this [up] and trying to transfer this technology to farmers,” Hernández Aguirre says. But he cautions that results obtained in laboratory conditions do not always transfer to the less controlled conditions found on the farm.

Both the Nottingham-CRC team and Hernández Aguirre are contending with the massive number of microbial species involved in the natural fermentation process. Even some of the seemingly minor players could have major roles in metabolizing compounds that may not otherwise be broken down into flavor molecules.

## Cutting out the microbes

Back in Switzerland, which has a legacy of producing premium chocolate, Chetschik is taking an unorthodox approach. “Fermentation is difficult to handle. It’s spontaneous and not easy to control,” she says. As a result, she has designed a process that is intended to produce robust chocolate flavors without the elaborate microbial networks of traditional fermentation.

The synthetic technique, which Chetschik calls “moist incubation,” starts with crushed, unfermented cacao beans. She simulates fermentation by heating the beans for 72 h in a slurry with lactic acid and ethanol, adding oxygen, and finally drying the resulting cocoa. “With the concentrations of ethanol that we have, the microbes can’t really grow,” she explains, so it’s faster and more manageable than natural fermentation.

Chocolate produced by moist incubation was tested by sensory panels, who reported that the aromas were fruitier, more flowery, maltier, and more caramel-like but less roasty than those of conventionally fermented cocoa. Panelists also reported fewer of the acidic notes that can occur with traditional fermentation.

Chetshik says that scaling up the simplified process should be feasible. CRC director Pathmanathan Umaharan says the method “may have a niche value to produce specialty chocolates.” But he points out that it’s unlikely the method would be economical for the 5 million t of cocoa produced annually, as heat must be put into the system if it isn’t produced naturally by microbes.

Chetschik’s work turns conventional cocoa fermentation on its head by shifting attention away from microbial metabolism in fine-flavor production. Instead, it puts a spotlight on protein hydrolysis—the breakdown of proteins by enzymes that are found naturally in cacao.

Once enzymes hydrolyze proteins, they leave behind peptides—short snippets of amino acids—but we still do not know everything about how peptides contribute to flavor, says Andrés Fernando González Barrios of the University of the Andes. In a recent metagenomic study of previously published data, González Barrios identified a greater diversity of peptides in fine-flavor chocolate; on average, those peptides were shorter than those found in chocolate made from bulk cocoa.

In an earlier study of fermentation temperature and peptide profiles, González Barrios showed that after the ferment is established, the beans experience a 4-day phase when their temperature remains high and largely constant. He suspects that the high temperature of that phase provides the right conditions for enzymatic hydrolysis and the creation of amino acids, dipeptides, and tripeptides.

But identifying which peptides have the biggest impact on flavor is not easy given the number of peptide sequences that enzymes can snip out of proteins, González Barrios says. “We need to carry out a lot of ‘peptidomics’ research in the future in order to see how those sequences are mapped with the sensory profile.”

## Back on the farm

In the end, González Barrios hopes that his research might help standardize how farmers carry out fermentation and improve the quality of chocolate.

Most cocoa farmers still rely on spontaneous fermentation, according to traditional local practices. But the CRC’s Ali says this can be attributed more to a lack of accessible information than to a lack of farmer’s curiosity. The Nottingham-CRC project “really triggered an interest in what is happening [during fermentation], and they are asking a lot more questions.” Ali is now preparing an easily understandable follow-up report for participating farms; in particular, she aims to explain the significance of the temperature and pH data the farmers collected and how they can use that information to follow the progression of fermentation.

Some farmers have already made small changes that are based on their studies’ findings. “Normally, we would just start the fermentation process, and there weren’t any scientific measurements,” Jagassar says. But now he plans to continue keeping tabs on the temperature and pH to help ascertain when the process is complete and drying should start.

**Figure:**
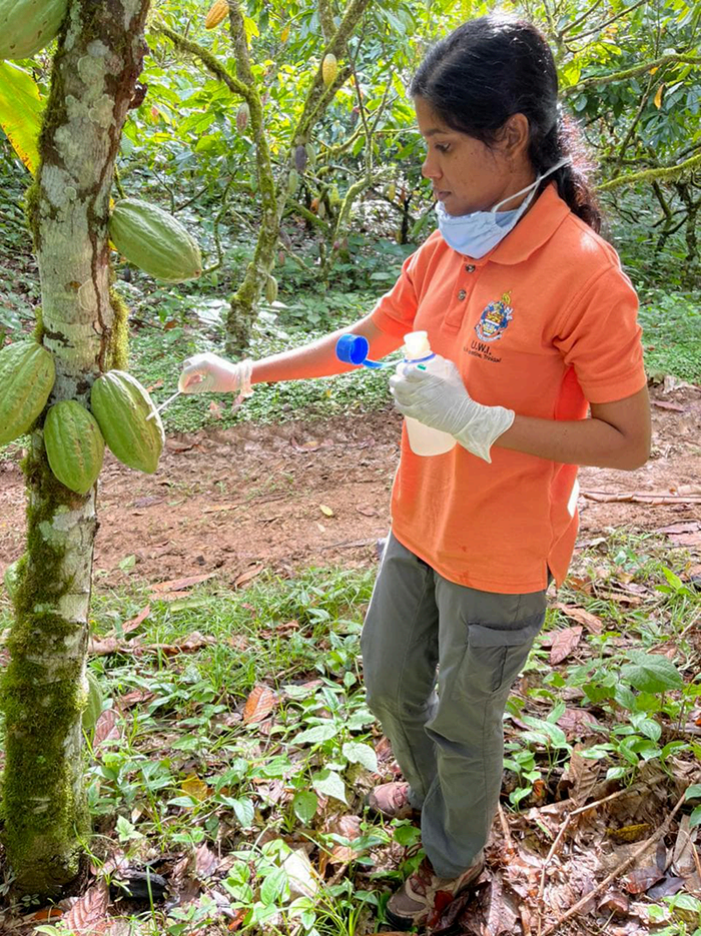
Naailah Ali swabs a pod as part of environmental microbe data gathering at San Juan Estate in Gran Couva, Trinidad and Tobago. Credit: Quincy Winklaar/San Juan Estate.

Trinidad farmer Charles Merry, whose cacao beans ranked among the top 50 in the world as part of the 2015 Cacao of Excellence Awards, also participated in the project. Based on the data the team collected, he says, his farm has now cut the traditional 7 fermentation days to 5 to avoid overfermenting.

Salt says one of the key things that has come out of the project so far is forming a connection between the farmers the researchers worked with in Colombia and the Nottingham-based bean-to-bar chocolate maker Luisa Vicinanza-Bedi, which has allowed the farmers to find a new market for their fine-flavor cocoa. Vicinanza-Bedi says the best of their chocolate provides long flavor journeys that include nut, red fruit, and lemon notes.

From the perspective of Vicinanza-Bedi and her business partner, Martyn O’Dare, these cacao farmers already have considerable tacit knowledge in controlling fermentation and adapting to various weather conditions. It “all takes skill, which is why bean-to-bar makers are so protective over their sources of ‘good cocoa’ and ensure the farmers are well paid for their excellent product.”

As consumers’ appetite for fine-flavor chocolate grows, the CRC’s Umaharan sees a future that will borrow from wine making, in which differences in grape varieties and local environments are combined with very precise control over the fermentation process, leading to a huge variety in flavor.

Chetschik agrees. “It is now starting to be the same for cacao. There’s a lot of promise, but a lot of work and research is needed.”

## Rachel Brazil is a freelance contributor to

Chemical & Engineering News, *the independent news outlet of the American Chemical Society*.

